# Ergodic Capacity Analysis of Full Duplex Relaying in the Presence of Co-Channel Interference in V2V Communications

**DOI:** 10.3390/s20010261

**Published:** 2020-01-02

**Authors:** Khaled Eshteiwi, Georges Kaddoum, M. S. Alam

**Affiliations:** ÉTS, LaCIME Laboratory, University of Québec, Montréal, QC H3C 1K3, Canada; georges.kaddoum@etsmtl.ca (G.K.); md-sahabul.alam.1@ens.etsmtl.ca (M.S.A.)

**Keywords:** full duplex relaying, Nakagami-m fading, ergodic capacity, co-channel interference, amplify-and-forward relaying

## Abstract

We analyze the ergodic capacity of a dual-hop full duplex amplify-and-forward (AF) vehicle-to-vehicle (V2V) cooperative relaying system over Nakagami-*m* fading channels. In this context, the impacts of self-interference (SI) at the relay and co-channel interference (CCI) at the destination are taken into account in this analysis. Precisely, based on the analysis of the moment generating function (MGF) of the signal-to-interference-plus-noise ratio (SINR), new exact and lower bound expressions for the ergodic capacity are derived. The ergodic capacity upper bound is also derived based on the asymptotic outage probability of the approximated SINR. Monte-Carlo simulation results are presented to corroborate the derived analytical results. Our results show the significant impact of the considered interferences on the system performance. It is shown that the ergodic capacity is degraded when the average SI at the relay and/or the average CCI at the destination is increased. This highlights the importance of taking these phenomena into account in the performance evaluation in order to assess the practical limit of full duplex relaying (FDR) cooperative wireless communications. Interestingly, it is also observed that FDR with SI and CCI still shows a higher ergodic capacity than the interference-free half duplex relaying, especially at medium to high signal-to-noise ratios (SNRs).

## 1. Introduction

Full duplex relaying techniques have recently become an interesting research area due to their ability to increase the spectral efficiency compared to half duplex relaying [1]. This increased efficiency is achieved because it allows the relays to avoid any spectral efficiency loss by transmitting and receiving over the same band simultaneously. However, the performance of full duplex relaying (FDR) systems is affected by the self-interference at the relay nodes which arises due to the simultaneous transmission and reception. Although the received signal is significantly weaker than the transmitted signal due to heavy path loss and fading [2,3], this effect cannot be perfectly eliminated. Meanwhile, in half duplex relaying (HDR), the relays are subject to a spectral efficiency loss as they transmit and receive in different time slots or over different frequency bands.

In the context of traditional HDR networks, the impact of co-channel interference (CCI) due to frequency reuse on the system performance has been extensively studied in the literature [4,5,6,7,8]. However, it has been noted that FDR networks, which are characterized by more frequency reuse, are more susceptible to CCI than their conventional HDR counterparts [9]. Therefore, in practical FDR protocol designs, the impact of CCI should be taken into account for a more accurate system analysis.

In this paper, we present a comprehensively analytical method in obtaining closed-form expressions for the exact, lower and upper bonds of ergodic capacity of V2V FDR cooperative wireless networks used AF relaying technique over i.n.i.d. Nakagami-m fading channels. Such closed form expressions are extremely desirable because they allow for prompt and adequate evaluation of the system performance. Monte-Carlo simulation results are presented to corroborate and validate the derived analytical results. Thus, giving an exact analysis for ergodic capacity of FDR which is valid for all SNR regimes is the main objective of this paper. To the best of our knowledge, the effect of CCI on full duplex (FD) with AF relaying over Nakagami-*m* fading channels has not been addressed in the available literature. We analyze the impact of CCI on FDR using variable gain AF relaying over independent but not necessarily identically distributed (i.n.i.d) Nakagami-*m* fading channels.

The main contributions of this paper are summarized below.

- We derive a closed form expression of the exact ergodic capacity of the equivalent end-to-end signal-to-interference-plus-noise ratio (SINR) of FD AF relaying over Nakagami-*m* fading channels under the effects of SI at the relay and of CCI at the destination.
- We also derive a closed form lower bound of the corresponding ergodic capacity. It should be noted that both the exact and the lower bound ergodic capacity expressions are derived based on the derivation of the SINR moment generating function (MGF) of the proposed system.
- We further derive a closed form expression for the ergodic capacity upper bound based on the asymptotic outage probability of the aforementioned system.

The remainder of this paper is organized as follows: Section 2 discusses the related work on ergodic capacity. Section 3 presents the system model. The exact, lower bound, and upper bound of the ergodic capacity expressions of the proposed system are derived in Section 4, and Section 5 presents the corresponding simulation results. Finally, closing remarks are discussed in Section 6.

## 2. Related Work

In this section, we present the works which mainly focus on the ergodic capacity analysis of FDR networks. FDR networks are often evaluated in terms of the ergodic capacity due to their ability to double the spectral efficiency compared to HDR. Recently, multiple-input multiple-output (MIMO) FDR techniques have attracted significant attention due to their ability to extend the network coverage, connectivity, and attain higher capacity without any requirement for extra power resources. Zero forcing (ZF) beamforming for MIMO FDR and HDR systems with CCI using amplify-and-forward (AF) relaying over Rayleigh fading channels was investigated in [9]. In [10], the impact of CCI at the relay node was investigated in terms of ergodic capacity for a one-way MIMO FDR system over Rayleigh fading channels. FDR systems with a non-orthogonal multiple access (NOMA) based communication system with two source–destination pairs were also investigated and analysed over Rayleigh fading channels in [11]. The impact of antenna correlation and imperfect channel state information (ICSI) on one-way MIMO FDR was analyzed in [12]. Similarly, the influence of antenna correlation on two-way massive MIMO FDR was considered in [13], where the achievable sum-rate was derived. The authors in [14] analyzed the spectral efficiency of massive MIMO FDR systems where the main channels and the self-interference (SI) channels follow Rayleigh and Rician distributions, respectively. In [15,16], the authors analyzed the ergodic capacity of FD cooperative NOMA employing the DF relaying protocol over Rayleigh fading channels. The authors in [17] presented the ergodic capacity of non-coherent MIMO Grassmannian modulation using DF over frequency flat block Rayleigh fading FDR. In [18], the authors evaluated the asymptotic ergodic capacity of AF FD relaying MIMO using Tracy–Widom distribution. In addition, they showed that increasing the number of source antennas can reduce the capacity, especially when the number of destination antennas is fixed. The authors in [19] studied the ergodic capacity of relay selection for two-way FD AF relaying over Rayleigh fading channels. In [20], the authors proposed FDR with joint antenna relay selection and SI elimination over block Rayleigh fading channels, where an ergodic capacity closed expression form was derived. The authors in [21] introduced the ergodic capacity of FDR for high speed railway using both DF and AF relaying over Rayleigh fading channels and compared its performance with HDR. In [22], the authors analyzed the upper bound ergodic capacity of two-way AF FDR of orthogonal frequency division multiplexing (OFDM) using physical layer network coding (PLNC). In [23], a two-way AF FDR over Rayleigh fading channels is considered, and the ergodic capacity closed form is provided where self-interference is simplified to be AWGN channel.

All of the above cited works were carried out assuming Rayleigh fading channels for ergodic capacity of FDR cooperative wireless networks. However, there are certain circumstances where the assumption of Rayleigh fading fails to represent the actual channel behavior of particular communication scenarios. For instance, if the suppression is not sufficient at the relay node, a line-of-sight (LOS) component may persist between the transmitter and receiver antennas at the relay and, hence, Rayleigh-fading is no longer a suitable model. Meanwhile, the Nakagami-*m* distribution is a more general model that can describe many fading distributions such as Rician and Rayleigh [24]. Furthermore, the Nakagami-*m* fading channel was shown to be the most accepted model for vehicular communications and short-range communications [25,26,27,28]. Motivated by these attributes, we consider the Nakagami-*m* fading channel model in this article.

Earlier works have mainly investigated the performance of FDR over Nakagami-*m* fading channels in terms of outage probability [29,30,31,32]. In [33], the authors studied the performance of FDR over Nakagami-*m* fading channels in terms of outage probability and asymptotic capacity. A similar performance analysis was carried out in [34] for FDR with decode-and-forward (DF) relaying, in which the impact of improper Gaussian signaling was investigated. Although instructive, these works were built upon the classical assumption that there is no CCI from adjacent cells.

## 3. System Model

**Figure 1:**
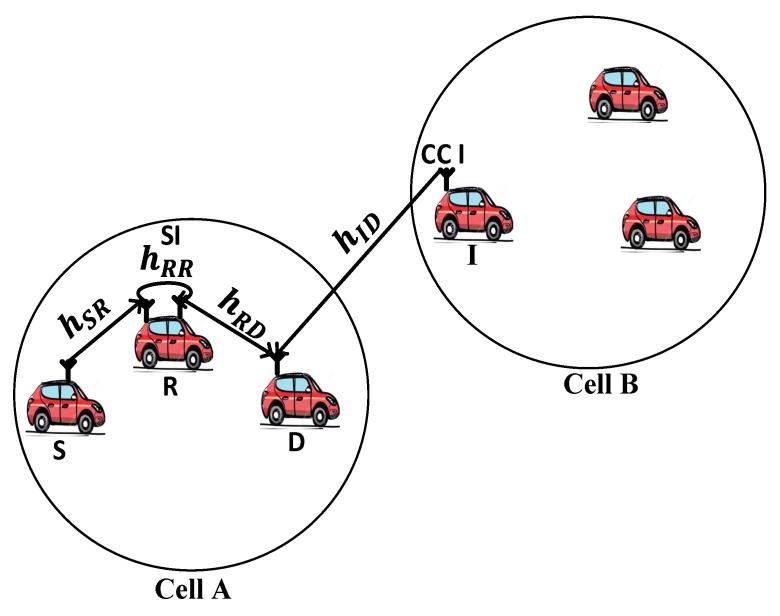
FDR with co-channel interference in vehicular cooperative wireless communications.

We consider a vehicle-to-vehicle (V2V) FDR scenario, where, in a single cell A, a source *S* communicates with a destination *D* via an AF relay *R*, as shown in Figure 1. It is assumed that the direct link between *S* and *D* is unavailable. In addition, we consider that *D* experiences CCI resulting from the spectrum re-usage of an adjacent user *I* in cell B. In this context, all the nodes are assumed to be equipped with a pair of antennas, one serving the purpose of transmitting while the other is for receiving. Thus, the received signal at *R* is corrupted by self-interference due to the simultaneous transmission/reception which characterizes the full duplex transmission mode.

In this vein, we list our system model assumptions below:

*Assumption 1*: The direct link between *S* and *D* is assumed absent. This is a realistic assumption in many cases especially when the distance between *S* and *D* is larger than the coverage of the source [35].

*Assumption 2*: In the proposed system model, full duplex relaying (FDR) networks, which are characterized by more frequency reuse, are more susceptible to co-channel interference (CCI) than their conventional half duplex relaying (HDR) counterparts [9]. Therefore, in practical FDR protocol designs, the impact of CCI should be taken into account for a more accurate system analysis.

*Assumption 3*: The Nakagami-*m* fading channel model is considered as it is widely used to model cooperative vehicular communication and short-range communications [25,26]. On the other hand, self-interference and its fading model heavily depend on the employed isolation/cancellation techniques. For instance, if the suppression is not sufficiently employed, line-of-sight (LOS) effects will persist and, hence, Rayleigh-fading will not be a suitable model since it does not count the LOS component. Moreover, the Nakagami-*m* fading is able to span a wide range of fading distributions that can also capture either scenarios in case of absence/presence of LOS effects. Furthermore, the Nakagami-*m* distribution is a more general model which can be used to describe many fading distributions such as Rician, Rayleigh or fading environment that is more severe than Rayleigh fading distribution [24]. Furthermore, the Nakagami-*m* fading channel was shown to be the most accepted model for vehicular communications and short-range communications [25,26,27,28]. A benefit of the Nakagami-m fading model is that it can be used when the received signal has contributions from both diffuse and specular scattering. It also offers greater flexibility and accuracy in fitting to experimental data [27]. Motivated by this, in this article, we considered the Nakagami-*m* fading channel model. We further assume that all underlying channels are quasi-static and remain constant during one slot, but change independently from one slot to another (this assumption is commonly used in the context of cooperative networks; see for example [36,37,38]) and the received signal undergoes slow fading (i.e., the symbol period of the received signal is smaller than the coherence time of the channel), which can be justified for V2V communications scenarios in rush-hour traffic.

In this context, the received signal at *R* is given as

(1) $$\begin{array}{cc} y_{SRR}= & \sqrt{P_{S}}h_{SR}x_{S}+\sqrt{P_{R}}h_{RR}x_{R}+n_{R}, \end{array}$$

where $x_{S}$ and $x_{R}$ are the transmitted binary phase shift keying (BPSK) signals while $P_{S}$ and $P_{R}$ denote the transmit powers at *S* and *R*, respectively. Moreover, $h_{SR}$ and $h_{RR}$ are the channel coefficients of the *S*-*R* and the self-interference *R*-*R* links, respectively. These are modeled as mutually i.n.i.d Nakagami-*m* random variables. All the channels are assumed to be quasi-static and remain constant during one slot, but change independently from one slot to another, which is typically the case for V2V communications scenarios in rush-hour traffic [36,37,38]. The term $n_{R}$ corresponds to the additive white Gaussian noise (AWGN) at *R* with variance $N_{o}$. At the destination side, the received signal from *R* is obtained as

(2) $$y_{D}=\sqrt{P_{R}}h_{RD}\kappa y_{SRR}+\sqrt{P_{I}}h_{ID}x_{I}+n_{D},$$

where $h_{RD}$ and $h_{ID}$ are the channel coefficients of the *R*-*D* (information link) and *I*-*D* (CCI link), respectively. In addition, $P_{I}$ is the transmitted power of the interference signal, $n_{D}$ is the AWGN at *D* with variance $N_{o}$, and κ is the amplification factor at *R* which satisfies $\kappa \leq \sqrt{P_{R}/(P_{S}{\left|h_{SR}\right|}^{2}+P_{R}{\left|h_{RR}\right|}^{2}+N_{o}}$ [28]. Here, perfect CSI is considered available at the destination node [25,39,40]. This assumption is valid since a feedback channel could be used to send pilot signals before the transmission to acquire knowledge about the channel properties [41]. In this study, we employ the normalized distance model [42,43,44] to take into account the impact of pathloss which implies that $E\left({\left|h_{ij}\right|}^{2}\right)={\left(d_{SD}/d_{ij}\right)}^{\eta }$, where $d_{ij}$ is the relative distance from *i* to *j*, $ij\in \left\{SR,RR,RD,ID\right\}$, and η is the pathloss exponent.

## 4. Performance Analysis

Considering the AF relaying strategy while taking into account the impact of SI at R and CCI at D, the instantaneous SINR of the received signal at *D* can be written as [45]

(3) $$\gamma_{eq}=\frac{\gamma_{1}\gamma_{2}}{\gamma_{1}+\gamma_{2}+1},$$

where $\gamma_{1}=\frac{\gamma_{SR}}{\gamma_{RR}+1}$ and $\gamma_{2}=\frac{\gamma_{RD}}{\gamma_{ID}+1}$. Here $\gamma_{SR}=P_{S}{\left|h_{SR}\right|}^{2}/N_{o}$, $\gamma_{RR}=P_{R}{\left|h_{RR}\right|}^{2}/N_{o}$, $\gamma_{RD}=P_{R}{\left|h_{RD}\right|}^{2}/N_{o}$, and $\gamma_{ID}=P_{I}{\left|h_{ID}\right|}^{2}/N_{o}$ denote the instantaneous SNRs of the *S*-*R*, *R*-*R*, *R*-*D* and *I*-*D* links, respectively. For the considered scenario, since the channel coefficients are modelled as Nakagami-*m* distributions, ${\left|h_{ij}\right|}^{2}$ follows a Gamma distribution. Moreover, since the channels are i.n.i.d, the PDF of $\gamma_{ij}$ can be written as [46]

(4) $$f_{\gamma_{ij}}\left(x\right)=\frac{C_{ij}^{m_{ij}}}{\Gamma (m_{ij})}x^{m_{ij}-1}\exp\left(-xC_{ij}\right),$$

where Γ(z)=$\int_{0}^{\infty }\exp\left(-t\right)t^{z-1}dt$ denotes the Gamma function [47] (Equation (8.310.1)), $m_{ij}>0.5$ is the shape parameter, $C_{ij}$=$\frac{m_{ij}}{{\bar{\gamma }}_{ij}}$, ${\bar{\gamma }}_{ij}$=$E\left({\left|h_{ij}\right|}^{2}\right)P_{i}/N_{o}$ is the average SNR, and E(.) is the expectation operator.

### 4.1. Exact Ergodic Capacity

The ergodic capacity is defined as the expected value of the instantaneous mutual information between the source and destination, which is given as

(5) $$\mathrm{EC}=E\left[\log_{2}\left(1+\gamma_{eq}\right)\right].$$

Substituting $\gamma_{eq}$ from Equation (3) into Equation (5), we get

(6) $$\mathrm{EC}=\frac{1}{\ln(2)}E\left[\ln\left(1+\frac{\gamma_{1}\gamma_{2}}{\gamma_{1}+\gamma_{2}+1}\right)\right].$$

**Theorem** **1.** *Assuming full duplex mode, the exact closed-form ergodic capacity expression of AF cooperative relaying systems with SI at the relay and CCI at the destination is given by Equation (7).*

(7) EC=1ln(2)∑s=0qqs(CSR)s∑w=0rrw(CRD)wΓ(q+r+2−s−w)(CRRmRRΓ(mRR)∑j=0mSR−1∑k=0j∑q=0∞jk(CSR)j−k−mRRj!×(k+mRR−1)!(−1)qq!∑f=0j+qj+qf(−1)j+q−f(CRRCSR)j+q−f(f−k−mRR+1)−(CRRCSR)f−k−mRR+1f−k−mRR+1)×(CIDmIDΓ(mID)∑l=0mRD−1∑t=0l∑r=0∞lt(CRD)l−t−mIDl!(t+mID−1)!(−1)rr!∑e=0l+rl+re(−1)l+r−e(CIDCRD)l+r−e×(e−t−mID+1)−(CIDCRD)e−t−mID+1e−t−mID+1).

**Proof.**  The proof is provided in Appendix A. □

### 4.2. Ergodic Capacity Lower Bound

To simplify the derivation of the ergodic capacity lower bound expression, let us re-write Equation (6) as follows

(8) $$\begin{array}{cc} \mathrm{EC} & =E\left[\log_{2}\left(\frac{\left(1+\gamma_{1}\right)\left(1+\gamma_{2}\right)}{\gamma_{1}+\gamma_{2}+1}\right)\right]\\ & ={\mathrm{EC}}_{\gamma_{1}}+{\mathrm{EC}}_{\gamma_{2}}-{\mathrm{EC}}_{\gamma_{T}}, \end{array}$$

where ${\mathrm{EC}}_{\gamma_{i}}$=$E[\log_{2}\left(1+\gamma_{i}\right)]$, $i\in \left\{1,2\right\}$, and ${\mathrm{EC}}_{\gamma_{T}}$=$E[\log_{2}\left(1+\gamma_{1}+\gamma_{2}\right)]$. Here, a lower bound for ${\mathrm{EC}}_{\gamma_{T}}$ is obtained by utilizing the Jensen’s inequality as follows

(9) $${\mathrm{EC}}_{\gamma_{T}}\leq \log_{2}\left(1+E\left(\gamma_{1}\right)+E\left(\gamma_{2}\right)\right).$$

**Theorem** **2.** *Assuming full duplex mode, the ergodic capacity lower bound of an AF cooperative relaying system with SI at the relay and CCI at the destination is given by Equation (10).*

(10) EClower=(1ln(2)CRRmRRΓ(mRR)∑j=0mSR−1∑k=0j∑q=0∞jk(CSR)j−k−mRRj!(k+mRR−1)!(−1)qq!∑f=0j+qj+qf(−1)j+q−f×(CRRCSR)j+q−f∑s=0qqs(CSR)q−sΓ(s+1)(f−k−mRR+1)−(CRRCSR)f−k−mRR+1f−k−mRR+1)+(1ln(2)CIDmIDΓ(mID)∑l=0mRD−1∑t=0l∑r=0∞lt(CRD)l−t−mIDl!(t+mID−1)!(−1)rr!∑e=0l+rl+re(−1)l+r−e×(CIDCRD)l+r−e∑u=0rru(CRD)r−uΓ(u+1)(e−t−mID+1)−(CIDCRD)e−t−mID+1e−t−mID+1)−log2(1+CSRmSRCRRmRRΓ(mSR)Γ(mRR)∑l1=0mSRmSRl1Γ(mRR+l1)CSRmRR+l1exp(CRR)∑l2=0mSR(−1)mSR−l2(CRRCSR)mSR−l2×(CSR)−(l2−mRR−l1+1)Γ(l2−mRR−l1+1,CRR)+CRDmRDCIDmIDΓ(mRD)Γ(mID)∑j1=0mRDmRDj1Γ(mID+j1)CRDmID+j1×exp(CID)∑j2=0mRD(−1)mRD−j2(CIDCRD)mRD−j2(CRD)−(j2−mID−j1+1)Γ(j2−mID−j1+1,CID)).

**Proof.**  The proof is provided in Appendix B. □

### 4.3. Ergodic Capacity Upper Bound

In order to provide further insights into the system performance, we also derive an ergodic capacity upper bound expression in the following theorem.

**Theorem** **3.** *Assuming full duplex mode, the ergodic capacity upper bound of AF cooperative relaying systems with SI at the relay and CCI at the destination is given by Equation (11), where $G\begin{array}{c} 1,1,1,1,1\\ 1,[1,1],1,[1,1] \end{array}\left(\begin{array}{c} \cdot \end{array}|\cdot \right)$ is the extended generalized bivariate Meijer’s G-function [48,49].*

(11) ECupper=CRRmRRCIDmIDln(2)Γ(mRR)Γ(mID)∑j=0mSR−1(CSR)jj!∑k=0jjk(k+mRR−1)!∑l=0mRD−1(CRD)ll!∑t=0llt(t+mID−1)!×∑q=0∞(−1)q(CSR+CRD)qq!Γ(k+mRR)CRRk+mRRΓ(t+mID)CIDt+mIDG1,1,1,1,11,[1,1],1,[1,1]−(j+l+q);(k+mRR);(t+mID)1+j+l+q;0;0|CSRCRR,CRDCID

**Proof.**  The proof is provided in Appendix C. □

## 5. Simulation Results

**Figure 2:**
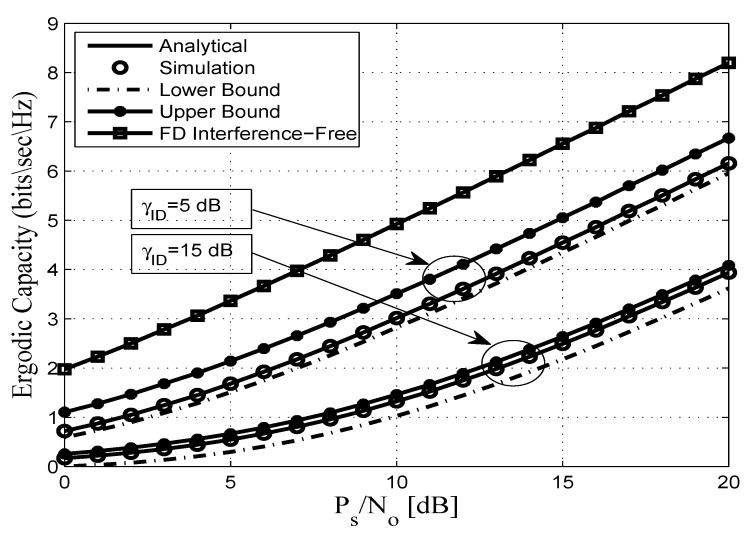
Ergodic capacity vs. $P_{S}/N_{o}$ for different values of ${\bar{\gamma }}_{ID}$ with $m_{ij}=2$, $d_{SR}=0.45$, $d_{RD}=0.55$, ${\bar{\gamma }}_{RR}=5$ dB, and η=3.18.

**Figure 3:**
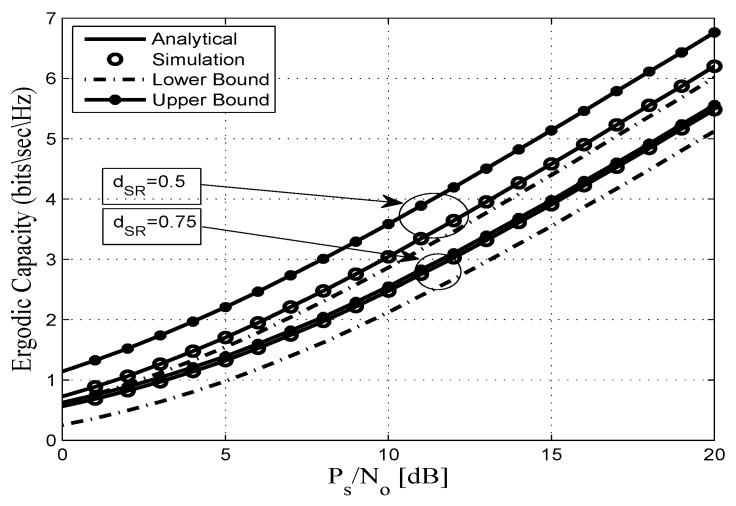
Ergodic capacity vs. $P_{S}/N_{o}$ for different values of $d_{SR}$ and $d_{RD}$ for ${\bar{\gamma }}_{ID}={\bar{\gamma }}_{RR}=5$ dB, $m_{ij}=2$, and η=3.18.

**Figure 4:**
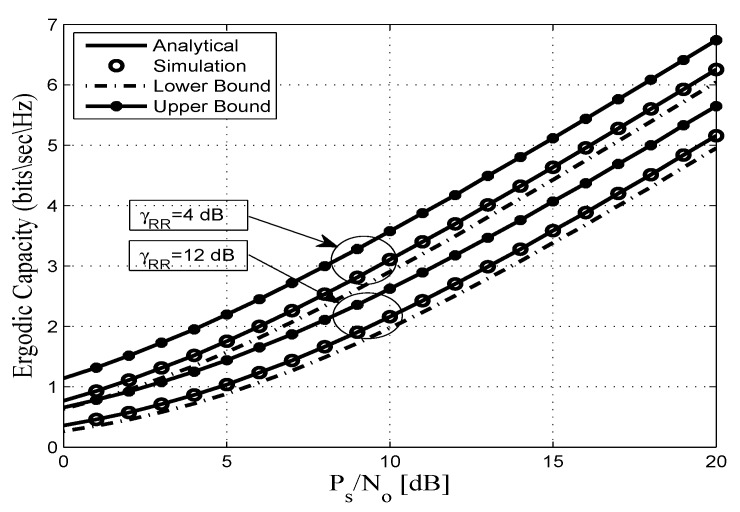
Ergodic capacity vs. $P_{S}/N_{o}$ for different values of ${\bar{\gamma }}_{RR}$ for $m_{ij}=2$, $d_{SR}=0.45$, $d_{RD}=0.55$, ${\bar{\gamma }}_{ID}=5$ dB, and η=3.18.

**Figure 5:**
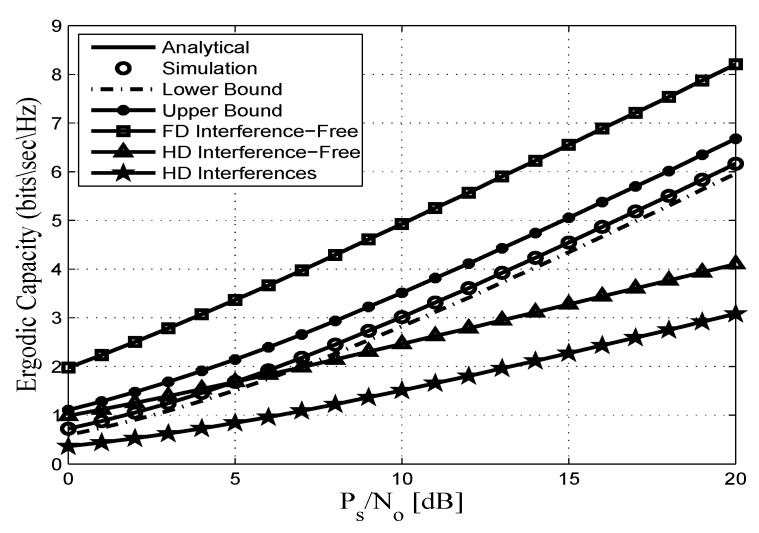
Ergodic capacity vs. $P_{S}/N_{o}$ for different relaying techniques with ${\bar{\gamma }}_{RR}={\bar{\gamma }}_{ID}=5$ dB, $m_{ij}=2$, $d_{SR}=0.45$, $d_{RD}=0.55$, and η=3.18.

**Figure 6:**
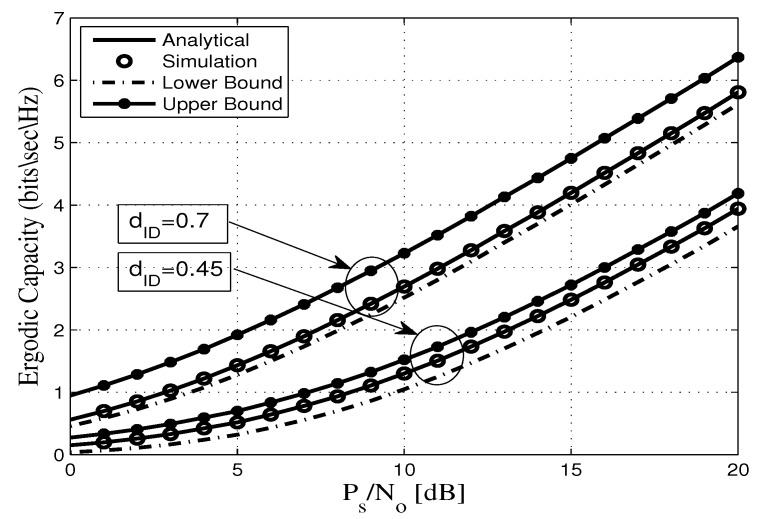
Ergodic capacity vs. $P_{S}/N_{o}$ for different values of $d_{ID}$ with ${\bar{\gamma }}_{RR}=8$ dB, $m_{ij}=2$, $d_{SR}=0.45$, $d_{RD}=0.55$, and η=3.18.

**Figure 7:**
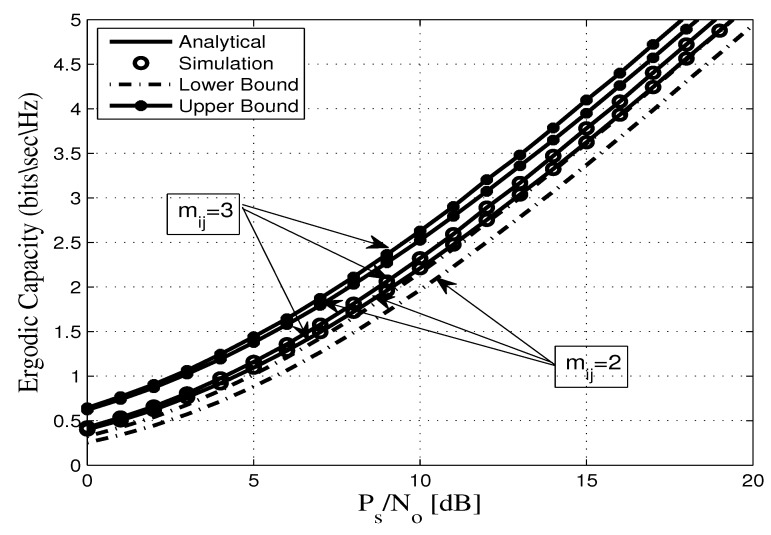
Ergodic capacity vs. $P_{S}/N_{o}$ for different values of $m_{ij}$ with ${\bar{\gamma }}_{RR}=5$ dB, ${\bar{\gamma }}_{ID}=10$ dB, $d_{SR}=0.45$, $d_{RD}=0.55$, and η=3.18.

This section assesses the accuracy of the derived analytical expressions and provides more insights into the performance of practical FDR networks. To this end, we consider an FDR network where the source communicates with the destination via an AF full duplex relaying including SI and CCI at the relay and destination nodes. Without any loss of generality all AWGN terms are assumed to be zero-mean complex Gaussian random variables with variance $N_{o}$. In addition, a pathloss exponent of η=3.18 and a shape parameter of $m_{ij}=2$ are considered. Unless otherwise stated, the derived analytical expressions are represented by solid lines, and the markers depict the corresponding simulation results. In this context, Figure 2, Figure 3, Figure 4, Figure 5, Figure 6 and Figure 7 show the exact as well as the lower and upper bounds of the ergodic capacity obtained in Equations (7), (10) and (11), respectively, where Monte Carlo simulation is utilized to validate the theoretical results. Here, a perfect match is observed between the analytical and the corresponding simulation results, while the derived lower and upper bound expressions provide a close approximation to the actual performance of the system.

Specifically, Figure 2 illustrates the ergodic capacity of the considered dual-hop FDR cooperative communication system versus the $P_{S}/N_{o}$ for different levels of CCI. The results show that when ${\bar{\gamma }}_{ID}$ goes from 5 dB to 15 dB, the upper bound becomes closer to the exact ergodic capacity while the lower bound is 0.5 dB lower than the exact value. Our results are compared to other FDR systems assuming that both the relay and the destination nodes are interference-free, which provides a significant ergodic capacity improvement. The simulation results in this figure are consistent with our theoretical analysis where the destructive impact of the co-channel interferences on the system performance are clearly indicated. For instance, in the ideal case (FD interference-free) the ergodic capacity = 5.6 bits/s/Hz at $P_{S}/N_{o}$ = 12 dB and for ${\bar{\gamma }}_{ID}$ = 5 dB, the corresponding ergodic capacity values are 3.6, 3.4, and 4.1 bits/s/Hz for the exact, lower, and upper bounds, respectively at $P_{S}/N_{o}$ = 12 dB, while the ergodic capacity values at ${\bar{\gamma }}_{ID}$ = 15 dB are 1.7, 1.4, and 1.9 bits/s/Hz for the exact, lower, and upper bounds, respectively. Hence, the considered interferences impose severe constraints on the system’s ergodic capacity, which highlights the need for both system characterization and the design of suitable interference cancellation techniques for the efficient implementation of the vehicular communication paradigm.

Figure 3 exhibits the performance of the proposed system in terms of ergodic capacity for different relay locations.This figure shows that the ergodic capacity of the system improves if the relay is placed in the middle of the source–destination pair (i.e., $d_{SR}$ = 0.5 and $d_{RD}$ = 0.5, with $d_{SD}$ = 1), which corresponds to a balanced average channel gain between the *S*–*R* and *R*–*D* links. However, the ergodic capacity is decreased when the relay is located close to the destination node ($d_{SR}$ = 0.75 and $d_{RD}$ = 0.25). For instance, for $d_{RD}$ = 0.5, at 10 dB, the exact, lower, and upper bound of the ergodic capacity values are 3, 2.8, and 3.6 bits/s/Hz, respectively. Meanwhile for $d_{RD}$ = 0.75, the corresponding exact, lower, and upper bound are 2.5, 2.1, and 2.6 bits/s/Hz, respectively.

Figure 4 shows the ergodic capacity of dual-hop FDR systems for different levels of self-interference at the relay. From the obtained results, it is clear that when the average self-interference at the relay increases, the system’s performance is deteriorated. For example, an ergodic capacity of 5.3 and 4.2 bits/s/Hz appears at 17 dB for ${\bar{\gamma }}_{RR}=$ 4 and ${\bar{\gamma }}_{RR}=$12 dB, respectively. This figure validates the theoretical results obtained in Equation (6), where the destructive influence of the self-interference on the ergodic capacity performance is eaisly observed. This can be explained by the fact that increasing the average SI at the relay degrades the relay transmitted signal to the destination node, which in turn will decreases the ergodic capacity, therefore affecting the system’s performance.

Figure 5 depicts the ergodic capacity of FDR and HDR in the presence/absence of interference. We observe that when there are no interferences at the relay and the destination nodes in both FDR and HDR schemes, FDR can achieve twice the capacity of HDR. For instance, when the SNR is around 20 dB, the ergodic capacity of the FDR system is 8.2 bits/s/Hz, whereas the ergodic capacity of the HDR is only 4.1 bits/s/Hz. This double capacity is achieved because the transmission and reception in HDR is accomplished in two time slots while only one time slot is enough for an FDR. In addition, the performances of both FDR and HDR are degraded in the presence of interferences at the relay and destination nodes. Interestingly, the figure also shows that FDR with SI and CCI still achieve higher ergodic capacity than to the interference free HDR case, especially at medium and high $P_{S}/N_{o}$.

Figure 6 plots the ergodic capacity as a function of the $P_{S}/N_{o}$ for different values of the distance between user *I* and the destination node ($d_{ID}$) where $P_{I}/N_{o}$ was set to 2 dB. It is clear that when the user *I* is close to the destination, the effective SNR between the user *I* and the destination increases, and consequently the ergodic capacity decreases, which degrades the system’s performance. For instance, for a target of ergodic capacity of 3 bits/s/Hz, the required transmit $P_{S}/N_{o}$ is 11 dB and 17 dB when $d_{ID}$=0.7 and 0.45, respectively.

As for the ergodic capacity, Figure 7 shows the ergodic capacity as a function of the $P_{S}/N_{o}$. The obtained results are compared to the exact simulation under different values of $m_{ij}$ in full duplex mode and under the impact of self-interference over Nakagami-*m* fading channels. It is clear that, as $m_{ij}$ increases, the ergodic capacity increases and the performance improves. Moreover, this figure clearly exhibits a perfect match between our analytical expressions and the corresponding simulation results. Meanwhile, it can be observed that the derived lower and upper bounds are close to the exact simulation results where a gap of approximately 1 dB is observed between the lower and upper bounds and the exact expression.

## 6. Conclusions

In this paper, we presented a comprehensive framework for the ergodic capacity evaluation of FDR in vehicular communications. Both SI at the relay and CCI at the destination were taken into account. In particular, we evaluated the performance of V2V wireless cooperative communications with an FD non-regenerative AF relaying system over i.n.i.d. Nakagami-*m* fading channels. Moreover, we derived closed form expressions for exact, lower bound, and upper bound expressions of the ergodic capacity. These expressions offer an efficient way to assess the ergodic capacity of the considered dual-hop FDR systems with SI and CCI. In this context, the influences of key system parameters such as the average CCI at the destination, the average SI at the relay node, and the relay position between the source and destination on the system performance were investigated. Our results showed that the system performance is significantly degraded by an increase in the average CCI at the destination or average SI at the relay. We Also found that the best location for the relay is in the middle of the source–destination link. Furthermore, we showed that when the user *I* is close to the destination node, the ergodic capacity decreases and the system’s performance degrades. This insight reveals the importance of taking into account these phenomena in order to provide pragmatic information with which to assess the practical limits of V2V FDR cooperative wireless communications to the system designer.

## Appendix A

Equation (6) can be written as [9]

(A1) $$\frac{1}{\ln(2)}E\left[\ln(1+\frac{\gamma_{1}\gamma_{2}}{\gamma_{1}+\gamma_{2}+1})\right]=\frac{1}{\ln(2)}\int_{0}^{\infty }\frac{1}{z}\left(1-M_{\gamma_{1}}\left(z\right)\right)\left(1-M_{\gamma_{2}}\left(z\right)\right)\exp\left(-z\right)dz,$$

where $M_{\gamma_{i}}\left(z\right)=E\left[\exp\left(-z\gamma_{i}\right)\right]$, $i\in \left\{1,2\right\}$. To solve Equation (A1), we need to derive both $M_{\gamma_{1}}\left(z\right)$ and $M_{\gamma_{2}}\left(z\right)$. Now, $M_{\gamma_{1}}\left(z\right)$ can be written as [50]

(A2) $$M_{\gamma_{1}}\left(z\right)=1-\int_{0}^{\infty }z\exp\left(-zx\right)\left[1-F_{\gamma_{1}}\left(x\right)\right]dx,$$

where $F_{\gamma_{1}}\left(x\right)$ is the cumulative distribution function (CDF) of $\gamma_{1}$ which can be evaluated as [51]

(A3) Fγ1(x)=1−CRRmRRΓ(mRR)exp(−xCSR)∑j=0mSR−1(xCSR)jj!∑k=0jjk(k+mRR−1)!(xCSR+CRR)k+mRR.

Substituting Equation (A3) into Equation (A2) and using [47] (Equation (1.211.1)), we have

(A4) $$M_{\gamma_{1}}\left(z\right)=1-z\vartheta \int_{0}^{\infty }\frac{x^{j+q}}{{\left(x+\frac{C_{RR}}{C_{SR}}\right)}^{k+m_{RR}}}dx,$$

where

(A5) ϑ=CRRmRRΓ(mRR)∑j=0mSR−1∑k=0j∑q=0∞jk(CSR)j−k−mRRj!(k+mRR−1)!(−1)qq!(z+CSR)q.

Taking g=x+u in Equation (A4), where $u=\frac{C_{RR}}{C_{SR}}$ and then using [47] (Equation (1.111)), $M_{\gamma_{1}}\left(z\right)$ can be expressed as

(A6) Mγ1(z)=1−zϑ∑f=0j+qj+qf(−1)j+q−f(CRRCSR)j+q−f(f−k−mRR+1)−(CRRCSR)f−k−mRR+1f−k−mRR+1.

Substituting ϑ from Equation (A5) into Equation (A6), yields

(A7) Mγ1(z)=1−zCRRmRRΓ(mRR)∑j=0mSR−1∑k=0j∑q=0∞jk(CSR)j−k−mRRj!(k+mRR−1)!(−1)qq!r×(z+CSR)q∑f=0j+qj+qf(−1)j+q−f(CRRCSR)j+q−f(f−k−mRR+1)−(CRRCSR)f−k−mRR+1f−k−mRR+1.

On the other hand, the CDF of $\gamma_{2}$ can be evaluated using [51]

(A8) Fγ2(x)=1−CIDmIDΓ(mID)exp(−xCRD)∑l=0mRD−1(xCRD)ll!∑t=0llt(t+mID−1)!(xCRD+CID)t+mID.

Following the same steps as for $M_{\gamma_{1}}\left(z\right)$, $M_{\gamma_{2}}\left(z\right)$ is obtained as

(A9) Mγ2(z)=1−zCIDmIDΓ(mID)∑l=0mRD−1∑t=0l∑r=0∞lt(CRD)l−t−mIDl!(t+mID−1)!(−1)rr!×(z+CRD)r∑e=0l+rl+re(−1)l+r−e(CIDCRD)l+r−e(e−t−mID+1)−(CIDCRD)e−t−mID+1e−t−mID+1.

Hence, substituting Equations (A7) and (A9) into Equation (A1) and after some mathematical manipulations, the proof is completed.

## Appendix B

From Equation (8), ${\mathrm{EC}}_{\gamma_{i}}=E\left[\log_{2}\left(1+\gamma_{i}\right)\right]=\frac{1}{\ln2}E\left[\ln\left(1+\gamma_{i}\right)\right]$ can be evaluated as [9]

(A10) $$E\left[\ln\left(1+\gamma_{i}\right)\right]=\int_{0}^{\infty }\frac{1}{z}\left(1-M_{\gamma_{i}}\left(z\right)\right)\exp\left(-z\right)dz,$$

where $M_{\gamma_{i}}\left(z\right)$, $i\in \left\{1,2\right\}$, is given in Equations (A7) and (A9). Here, Equation (A10) is valid for any $\gamma_{i}\geq 0$ [9]. Now, substituting the value of $M_{\gamma_{1}}\left(z\right)$ from Equation (A7) into Equation (A10), and utilizing [47] (Equations (1.111) and (3.351.3)), we have

(A11) ECγ1=1ln2CRRmRRΓ(mRR)∑j=0mSR−1∑k=0j∑q=0∞jk(CSR)j−k−mRRj!(k+mRR−1)!(−1)qq!∑f=0j+qj+qf(−1)j+q−f×(CRRCSR)j+q−f∑s=0qqs(CSR)q−sΓ(s+1)(f−k−mRR+1)−(CRRCSR)f−k−mRR+1f−k−mRR+1.

Similarly,

(A12) ECγ2=1ln2CIDmIDΓ(mID)∑l=0mRD−1∑t=0l∑r=0∞lt(CRD)l−t−mIDl!(t+mID−1)!(−1)rr!∑e=0l+rl+re(−1)l+r−e×(CIDCRD)l+r−e∑u=0rru(CRD)r−uΓ(u+1)(e−t−mID+1)−(CIDCRD)e−t−mID+1e−t−mID+1

Since, the probability density function (PDF) of $\gamma_{1}$ is given by [52]

(A13) fγ1(y)=CSRmSRCRRmRRymSR−1Γ(mSR)Γ(mRR)exp(−yCSR)∑l1=0mSRmSRl1Γ(mRR+l1)(yCSR+CRR)mRR+l1

We can evaluate $E[\left(\gamma_{1}\right)]$ and $E[\left(\gamma_{2}\right)]$ in Equation (9) using Equation (A13), as follows

(A14) E[(γ1)]=CSRmSRCRRmRRΓ(mSR)Γ(mRR)∑l1=0mSRmSRl1Γ(mRR+l1)CSRmRR+l1∫0∞ymSRexp(−yCSR)(y+CRRCSR)mRR+l1dy︸I1.

For the integral $I_{1}$, taking w=y+u with $u=\frac{C_{RR}}{C_{SR}}$ and using the binomial expansion [47] (Equation (1.111)), we get

(A15) $$I_{1}=\exp\left(C_{RR}\right)\sum_{l_{2}=0}^{m_{SR}}{\left(-1\right)}^{m_{SR}-l_{2}}{\left(\frac{C_{RR}}{C_{SR}}\right)}^{m_{SR}-l_{2}}\int_{u}^{\infty }w^{l_{2}-m_{RR}-l_{1}}\exp\left(-wC_{SR}\right)dw.$$

Utilizing [47] (Equation (3.381.3)), $I_{1}$ is obtained as

(A16) $$I_{1}=\exp\left(C_{RR}\right)\sum_{l_{2}=0}^{m_{SR}}{\left(-1\right)}^{m_{SR}-l_{2}}{\left(\frac{C_{RR}}{C_{SR}}\right)}^{m_{SR}-l_{2}}{\left(C_{SR}\right)}^{-(l_{2}-m_{RR}-l_{1}+1)}\Gamma \left(l_{2}-m_{RR}-l_{1}+1,C_{RR}\right)$$

Inserting Equation (A16) into Equation (A15) and substituting $I_{1}$ into Equation (A14), we get

(A17) E[(γ1)]=CSRmSRCRRmRRΓ(mSR)Γ(mRR)∑l1=0mSRmSRl1Γ(mRR+l1)CSRmRR+l1exp(CRR)∑l2=0mSR(−1)mSR−l2×(CRRCSR)mSR−l2(CSR)−(l2−mRR−l1+1)Γ(l2−mRR−l1+1,CRR).

Furthermore, the PDF of $\gamma_{2}$ can be written as [52]

(A18) fγ2(y)=CRDmRDCIDmIDymRD−1exp(−yCRD)Γ(mRD)Γ(mID)∑j1=0mRDmRDj1Γ(mID+j1)(yCRD+CID)mID+j1.

Following the same steps, $E[\left(\gamma_{2}\right)]$ is obtained as

E[(γ2)]=CRDmRDCIDmIDΓ(mRD)Γ(mID)∑j1=0mRDmRDj1Γ(mID+j1)CRDmID+j1exp(CID)∑j2=0mRD(−1)mRD−j2×(CIDCRD)mRD−j2(CRD)−(j2−mID−j1+1)Γ(j2−mID−j1+1,CID).

Finally, substituting Equations (A11), (A12), (A17) and (A19) into Equation (8) yields Equation (10).

## Appendix C

The upper bound of the ergodic capacity can be evaluated as [33]

(A19) $${\mathrm{EC}}_{upper}=\frac{1}{\ln2}\int_{0}^{\infty }\frac{1-F_{\gamma_{eq}}\left(x\right)}{1+x}dx,$$

where

(A20) $$\begin{array}{cc} F_{\gamma_{eq}}\left(x\right) & =\mathrm{Pr}[\min\left(\gamma_{1},\gamma_{2}\right)\leq x]\\ & =1-\left(1-F_{\gamma_{1}}\left(x\right)\right)\left(1-F_{\gamma_{2}}\left(x\right)\right). \end{array}$$

Substituting Equations (A3) and (A8) into Equation (A21), we get

(A21) Fγeq(x)=1−CRRmRRΓ(mRR)exp(−xCSR)∑j=0mSR−1(xCSR)jj!∑k=0jjk(k+mRR−1)!(xCSR+CRR)k+mRR×CIDmIDΓ(mID)exp(−xCRD)∑l=0mRD−1(xCRD)ll!∑t=0llt(t+mID−1)!(xCRD+CID)t+mID.

Substituting Equation (A22) into Equation (A20), we get

(A22) $${\mathrm{EC}}_{upper}=\frac{A}{\ln2}\int_{0}^{\infty }x^{j+l}\exp\left[-x\left(C_{SR}+C_{RD}\right)\right]\frac{1}{1+x}\frac{1}{{\left(xC_{SR}+C_{RR}\right)}^{k+m_{RR}}}\frac{1}{{\left(xC_{RD}+C_{ID}\right)}^{t+m_{ID}}}dx,$$

where

(A23) A=CRRmRRΓ(mRR)∑j=0mSR−1(CSR)jj!∑k=0jjk(k+mRR−1)!CIDmIDΓ(mID)∑l=0mRD−1(CRD)ll!∑t=0llt(t+mID−1)!

Now, our task is to solve the integral in Equation (A23). Given the following identity

(A24) $${\left(\frac{1}{1+\upsilon x}\right)}^{\beta }=\frac{1}{\Gamma (\beta )}G\begin{array}{c} 1,1\\ 1,1 \end{array}\left(\begin{array}{c} 1-\beta \\ 0 \end{array}|\upsilon x\right),$$

Equation (A23) can be rewritten as

(A25) $$\begin{array}{cc} {\mathrm{EC}}_{upper}= & \;\frac{A}{\ln2}\sum_{q=0}^{\infty }\frac{{\left(-1\right)}^{q}{\left(C_{SR}+C_{RD}\right)}^{q}}{q!}\frac{1}{\Gamma \left(k+m_{RR}\right)C_{RR}^{k+m_{RR}}\Gamma \left(t+m_{ID}\right)C_{ID}^{t+m_{ID}}}\\ \; & \times \int_{0}^{\infty }x^{j+l+q}G\begin{array}{c} 1,1\\ 1,1 \end{array}\left(\begin{array}{c} 0\\ 0 \end{array}|x\right)G\begin{array}{c} 1,1\\ 1,1 \end{array}\left(\begin{array}{c} 1-(k+m_{RR})\\ 0 \end{array}|\frac{C_{SR}}{C_{RR}}x\right)G\begin{array}{c} 1,1\\ 1,1 \end{array}\left(\begin{array}{c} 1-(t+m_{ID})\\ 0 \end{array}|\frac{C_{RD}}{C_{ID}}x\right)dx. \end{array}$$

The second and third Meijer’s G-functions in the right hand side (R.H.S) of Equation (A26) are transformed into an extended generalized bivariate Meijer’s G-function according to the functional relation in [5]. Making use of [48] (4.1) and then inserting Equation (A24) into Equation (A26), yields (11) which concludes the proof.

It should also be noted that the extended generalized bivariate Meijer’s G-function $G\begin{array}{c} \beta ,\epsilon ,\varepsilon ,\zeta ,\nu \\ \Delta ,[\Theta ,\Omega ],\Upsilon ,[\Phi ,\Psi ] \end{array}\left(\begin{array}{c} a_{1},\ldots ,a_{\Delta };b_{1},\ldots ,b_{\Theta };c_{1},\ldots ,c_{\Omega }\\ e_{1},\ldots ,e_{\Upsilon };f_{1},\ldots ,f_{\Phi };g_{1},\ldots ,g_{\Psi } \end{array}|z_{1},z_{2}\right)$ converges based on two conditions as mentioned in [53]: Δ+Φ+Υ+Θ<2(ζ+β+ϵ) and Δ+Ψ+Υ+Ω<2(ν+β+ε). It is straightforward to show that the parameters of the extended generalized bivariate Meijer’s G-function in Equation (11) satisfy these two conditions, and therefore the Meijer’s G-functions converge.
